# Abnormal Expression of Cerebrospinal Fluid Cation Chloride Cotransporters in Patients with Rett Syndrome

**DOI:** 10.1371/journal.pone.0068851

**Published:** 2013-07-19

**Authors:** Sofia Temudo Duarte, Judith Armstrong, Ana Roche, Carlos Ortez, Ana Pérez, Maria del Mar O’Callaghan, Antonina Pereira, Francesc Sanmartí, Aida Ormazábal, Rafael Artuch, Mercedes Pineda, Angels García-Cazorla

**Affiliations:** 1 Department of Neurology, Hospital Sant Joan de Déu (HSJD), Barcelona, Spain; 2 Department of Biochemistry, Hospital Sant Joan de Déu (HSJD), Barcelona, Spain; 3 CIBER-ER (Biomedical Network Research Centre on Rare Diseases, Instituto de Salud Carlos III), Madrid, Spain; 4 Instituto de Medicina Molecular, Faculdade de Medicina da Universidade de Lisboa, Lisboa, Portugal; 5 Instituto Gulbenkian de Ciência, Oeiras, Portugal; Institute of Genetics and Biophysics, Italy

## Abstract

**Objective:**

Rett Syndrome is a progressive neurodevelopmental disorder caused mainly by mutations in the gene encoding methyl-CpG-binding protein 2. The relevance of MeCP2 for GABAergic function was previously documented in animal models. In these models, animals show deficits in brain-derived neurotrophic factor, which is thought to contribute to the pathogenesis of this disease. Neuronal Cation Chloride Cotransporters (CCCs) play a key role in GABAergic neuronal maturation, and brain-derived neurotrophic factor is implicated in the regulation of CCCs expression during development. Our aim was to analyse the expression of two relevant CCCs, NKCC1 and KCC2, in the cerebrospinal fluid of Rett syndrome patients and compare it with a normal control group.

**Methods:**

The presence of bumetanide sensitive NKCC1 and KCC2 was analysed in cerebrospinal fluid samples from a control pediatric population (1 day to 14 years of life) and from Rett syndrome patients (2 to 19 years of life), by immunoblot analysis.

**Results:**

Both proteins were detected in the cerebrospinal fluid and their levels are higher in the early postnatal period. However, Rett syndrome patients showed significantly reduced levels of KCC2 and KCC2/NKCC1 ratio when compared to the control group.

**Conclusions:**

Reduced KCC2/NKCC1 ratio in the cerebrospinal fluid of Rett Syndrome patients suggests a disturbed process of GABAergic neuronal maturation and open up a new therapeutic perspective.

## Introduction

Rett syndrome (RTT) is an X-linked neurodevelopmental disorder with an incidence of 1∶10000 live female births and is one of the leading causes of mental retardation and autistic behavior in females [1]. Loss-of-function mutations in the gene encoding methyl-CpG binding protein 2 (MeCP2) cause most cases of RTT. Individuals affected with RTT experience normal development up to the age of 6–18 months, at which time they fail to acquire new skills and enter a period of motor regression [2]. Autistic features are a hallmark of this disorder and epilepsy is frequent [3]. RTT patient brain does not show obvious signs of neurodegeneration, atrophy, gliosis, demyelination, or neuronal migration defects [4], [5], suggesting that neurological symptoms may primarily stem from subtle defects of subcellular compartments such as dendrites, axons, or synaptic structures [6]. MeCP2 is a transcriptional regulatory protein, and in its absence, a large number of genes exhibit abnormal expression with implications in the balance between synaptic excitation and inhibition [7], [8]. MeCP2 might be particularly important to GABAergic function and there is evidence that the expression of MeCP2 is approximatelly 50% higher in GABAergic neurons when compared to non GABAergic neurons. Mice with conditional deletion of *Mecp2* in GABAergic neurons initially show normal behavior but in the course of development start displaying forepaw stereotyped movements, compulsive grooming, impaired motor coordination, learning/memory deficits, abnormal EEG hyperexcitability, severe respiratory dysrhythmias and premature lethality [8].

γ-aminobutyric acid (GABA) is the main inhibitory neurotransmitter in the adult brain. During early development, activation of the chloride- permeable, postsynaptic, GABAA receptors (GABAA-R) can induce depolarization and the basal intracellular chloride concentration is determinant for the action of GABA in the developing neurons [9]. Two major contributors to intracellular chloride concentration are NKCC1 (Na^+^, K^+^, 2Cl^−^ cotransporter, that accumulates chloride in the cell), and KCC2 (K^+^, Cl^−^ cotransporter, that extrudes chloride). Several lines of research correlate epileptogenesis with altered function of NKCC1 and KCC2 [10], [11]. *In vitro*, experiments suggest that bumetanide, a potent NKCC1 inhibitor, can increase GABAergic inhibition, in combination with phenobarbital [12]. Bumetanide has also been reported useful in a neonatal patient with seizures [13] and in autistic children [14].

Moreover, the brain of MeCP2 deficient animal models shows deficits in brain-derived neurotrophic factor [15] (BDNF), which is thought to contribute to the pathogenesis of RTT. BDNF can also promote the functional maturation of GABAA-R mediated responses by inducing upregulation of KCC2 [16], [17], [18].

Human age related changes in GABAA-R physiology remain controversial, although neuropathological studies have already identified postnatal developmental changes of NKCC1 and KCC2 cortical expression [18]. The detection of synaptic proteins in the cerebrospinal fluid (CSF) gives us the possibility to indirectly access synaptic composition and alterations, using the CSF of patients with disorders related to neurotransmission, with the advantage of performing these studies *in vivo* [19].

We hypothesize that changes in BDNF expression levels or the direct effect of the underlying genetic mutation can interfere with the normal expression of NKCC1 and KCC2 leading to a reduction in the KCC2/NKCC1 ratio, characteristic of the immature GABAergic system. A comparison of NKCC1 and KCC2 protein levels in the CSF of patients affected with RTT and a control population was made in order to address this question.

## Patients and Methods

### Patients and Controls

Sixteen patients with RTT were recruited to this study, aged between 2 to 19 years at the moment of CSF collection. Patients’ clinical characteristics are summarized in table 1. Patients without a documented mutation fulfilled clinical criteria for RTT according to the last updated revision [20]. The control study was performed in 67 subjects (age range: 1 day - 14 years; mean: 740 days; female: 27; male: 40) whose CSF samples were submitted to Hospital San Joan de Deu (HSJD) laboratory under suspicion of viral or bacterial meningitis or encephalitis. Exclusion criteria were: diagnosis of viral or bacterial meningitis, neurologic disease, and hematic or xantocromic CSF (blood contamination).

**Table 1:** Clinical and laboratory features of Rett Syndrome patients included in the study.

	Age (Yearsat CSF collection)	Genetic screening	Epilepsy	Medication (when LP was performed)	Respiratory anomalies	KCC2/NKCC1 (optical densities)
1	2	MECP2 screened, no alteration found	No	NO AED	No	36795/310023
2	2	P.Y141X	Refractory EpilepsyGeneralized seizures	NO AED	No	0/242968
3	2	P.R270X	Refractory epilepsy	No AED	Severe syperventilation bursts and apneas	0/323889
4	4	P302H, 905C>T	Generalized seizures from 2 years of life	No AED	Hyperventilation bursts	167071/228307
5	5	P.R306C	Epileptic status Generalized seizures from 4 years of life	VPA	Hyperventilation bursts and apneas	0/169395
6	6	MECP2, CDKL5 screened, noalteration found	Generalized seizures since 8 years of life	CBZ	Hyperventilation bursts	177102/257110
7	7	MECP2, CDKL5 screened, noalteration found	Reflex seizures AbcencesAtonic seizures	No AED	No	28646/296735
8	8	P.R255X	Generalized seizures from2 years of life	VPA	Hyperventilation bursts and apneas	15584/342592
9	9	MECP2, CDKL5 screened, noalteration found	Refractory epilepsy	VPA TPM	Severe Hyperventilation and apneas	0/54010
10	10	P.R306C	Absences and partial seizures from 8 years	CBZ	Hyperventilation bursts	155391/621440
11	11	DEL EX.1–2	Generalized seizures from 8 years	No AED	Hyperventilation	91959/527745
12	16	MECP2, CDKL5 screened, noalteration found	Generalized seizures from 11 years of life	No AED (VPA was withdrawn 2 yearsbefore LP)	Hyperventilation	156883/445649
13	16	MECP2, CDKL5screened, no alteration found. Polymorfism in NTNG1	Generalized and absence seizures from 14 years of life	VPA CBZ	Hyperventilation bursts	164531/259927
14	16	P.R294X	Generalized Seizures from 6 years of life	CBZ LEV	Hyperventilation bursts	219795/281395
15	18	MECP2, CDKL5 screened, noalteration found	Partial seizures	CBZ	Hyperventilation	7000/535292
16	19	P.R294X	Partial, secondarilygeneralized and absences	CBZ	Hyperventilation bursts and apneas	604155/28867

DEL: Deletion. LP: Lumbar Puncture. AED: Anti epileptic drugs. VPA: Valproic Acid. CBZ: Carbamazepine. LEV: Levetiracetam. TPM: Topiramate.

### CSF Samples

CSF samples were collected by lumbar puncture as previously described [21]. They were obtained after parent’s written informed consent and in accordance with the Helsinki Declaration of 1964, as revised in 2000. The ethical committee of HSJD approved the study. After lumbar puncture, the first ten drops were used for routine cytochemical/microbiological studies and then CSF was immediately stored in 4 aliquots at −80°C until the moment of analysis. Biogenic amines metabolites and synaptic proteins were studied using the following 20 drops.

NKCC1 and KCC2 expression levels were analyzed by western blot. Twenty µL of CSF were loaded on gel and proteins were separated on a 10% sodium dodecyl sulphate-polyacrylamide gel and transferred to polyvinylidene difluoride membrane (AmershamTM HybondTM –ECL, GE Healthcare). Membranes were blocked in TBST buffer (0.02 M Tris-base, pH7.6, 0.8% NaCl, 0.1% Tween 20) with 5% dry skimmed milk for 60 min at room temperature. Anti-NKCC1 (1∶500; Santa Cruz Biotechnology®) and anti-KCC2 (1∶500; Millipore®) antibodies were added and incubated at 4 °C overnight. Membranes were washed three times with TBST buffer followed by incubation with appropriate anti-rabbit (1∶3000, Promega®) IgG secondary antibody at room temperature for 1 h. The blot was then washed six times with TBST and signal was revealed with ECL (Pierce® ECL Western Blotting Substract, Thermo Scientific). Relative levels of each protein were quantified by measuring optical densities (OD) of the corresponding bands with Quantity One® V 4.3.1.software.

### Statistical Analysis

Statistical analysis was performed using IBM Statistical Package for the Social Sciences (IBM SPSS Statistics Version 19.0, SPSS Inc: Chicago, IL). A significance level of.05 was used in all analyses. Outlier analysis was done taking into account the primary variable in the study – healthy/RTT. Outliers (defined as values 1.5 times lower than the 1st quartile or 1.5 times higher than the 3rd quartile) [22] were found in 8 cases (1 Rett patient, 7 healthy controls) for KCC2/NKCC1 ratio, 4 cases for NKCC1 (1 Rett patient, 3 healthy controls) and in 1 case (healthy control) for KCC2. Outliers were excluded from the respective analyses.

Non-parametric tests were applied when possible for age, NKCC1 and KCC2, since the assumption of normal distribution was not fulfilled for these variables.

## Results

**Figure 1:**
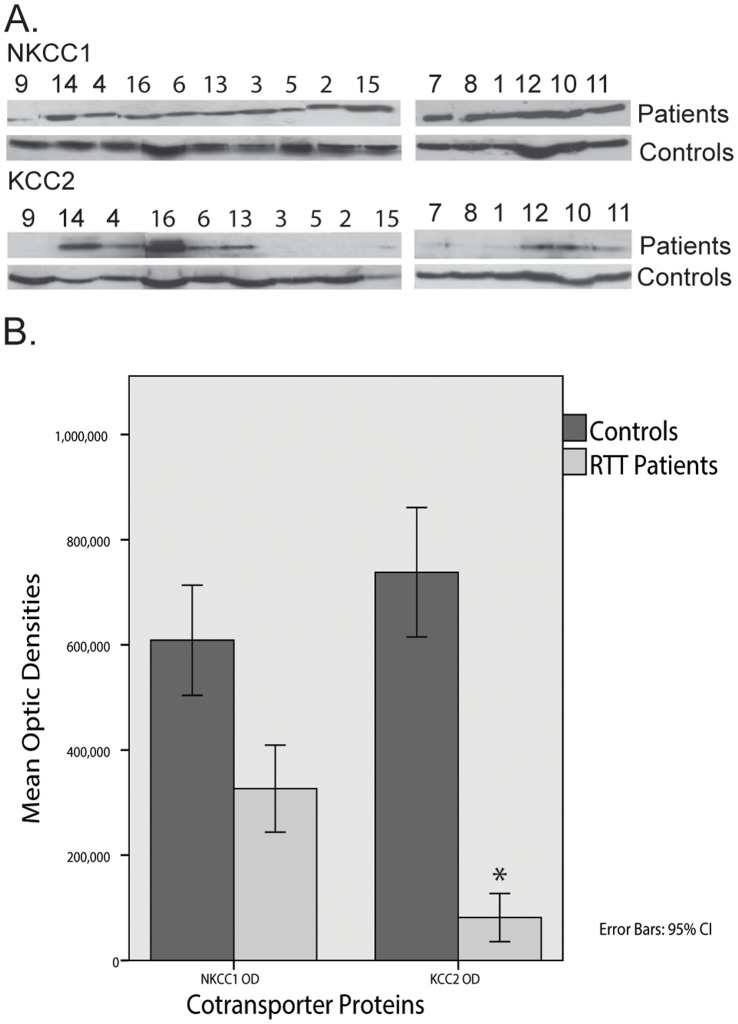
NKCC1 and KCC2 Cotransporters in the CSF of Rett Syndrome (RTT) Patients and Controls. (A) Immunoblot results in RTT patients and controls. Numbers refer to the patient ID numbers of Table 1. Comparison of patients and age matched controls. (B) Mean Optic Densities of NKCC1 and KCC2 Cotransporter Proteins for Rett Patients and Controls suggesting discrepant cotransporter levels between Patients and Controls supported by the respective MANCOVA (*F* (1, 73) = 6.99, *p*<.01, *η_p_^2^* = .087). Error bars represent 95% Confidence Interval. *p<.01.

Total CSF protein concentration values (*M = *33.27, *SD* = 19.04, range: 7–73 g/l) were within normal limits according to different age ranges [23]. Clinical and genetic features of RTT patients are described in table 1. NKCC1 and KCC2 western blot analysis were performed on the CSF of controls and RTT patients. CCCs were detected in the CSF of this population, at the expected molecular weight (Figure 1A). Considering the reported sexually dimorphic expression of KCC2 and GABA function in the substantia nigra [24] it was decided to control for gender in all reported analysis.

Furthermore, the homogenous distribution of the demographical variable age was verified with Mann-Whitney Test (*U*). Controls (*M = *746.00 days, *SD* = 1075.89) were significantly (*U* = 932.00, *p*<.001) younger than RTT patients (*M* = 3444.69 days, *SD* = 2173.59) therefore we have controlled for this variable in all reported analysis (table S1).

### KCC2 Expression is Decreased in the CSF of Rett Syndrome Patients

As patients grew older their OD signal of KCC2 in the CSF decreased. Partial correlation analysis was performed to clarify the relationship between age and the OD signal of the different synaptic proteins (NKCC1, KCC2) in each group (RTT, Controls), controlling for the effect of gender. A negative correlation between age and the OD signal of KCC2 (*r* (74) = −.292, n = 80, *p*<.05) has been clearly identified in our data, i.e. as the participants grew older their OD signal of KCC2 decreased, even when controlling for the effect of gender. Meaning that, KCC2 levels decrease in CSF throughout aging. However, concerning the OD signal of NKCC1 this correlation did not reach statistical significance (*r* (74) = −.207, *p*>.05). Interestingly, if the effect of gender was not taken into consideration, a strong, negative correlation between age and the OD signal of KCC2 (*r_s_* (80) = −.509, *p*<.0001) and NKCC1 (*r_s_* (78) =  −.472, *p*<.0001) would have been identified in our data, i.e. as the participants grew older their OD signals of KCC2 and NKCC1 decrease.

### Rett Patients Present a Significantly Lower OD Signal of KCC2 than Healthy Controls (Figure 1B)

To determine whether there were statistically significant differences in the OD signal of the different synaptic proteins (NKCC1, KCC2) between healthy controls and RTT patients, a between-subjects MANCOVA was performed, controlling for the effects of age and gender. This analysis revealed a statistically significant difference between healthy controls and RTT patients, *F* (1, 73) = 6.99, *p*<.01, *η_p_^2^* = .087 even when controlling for the possible confounding effects of age and gender. The subsequent follow-up Univariate ANCOVAs run to specify the characteristics of this finding revealed that RTT patients presented a significantly lower OD signal of KCC2 (*M* = 81383.80, *SD* = 82370.99; *F* (1, 76) = 12.28, *p<*.001, *η_p_^2^* = .139) than healthy controls (*M* = 692663.52, *SD* = 425753.91). Notwithstanding, NKCC1 expression does not differ significantly between patients (*M* = 307834.25, *SD* = 162592.59) and healthy controls, (*M* = 608703.27, *SD* = 416157.33; *F* (1, 74) = 1.87, *p*>.05).

### KCC2/NKCC1 Ratio is Decreased in the CSF of Rett Syndrome Patients

**Figure 2:**
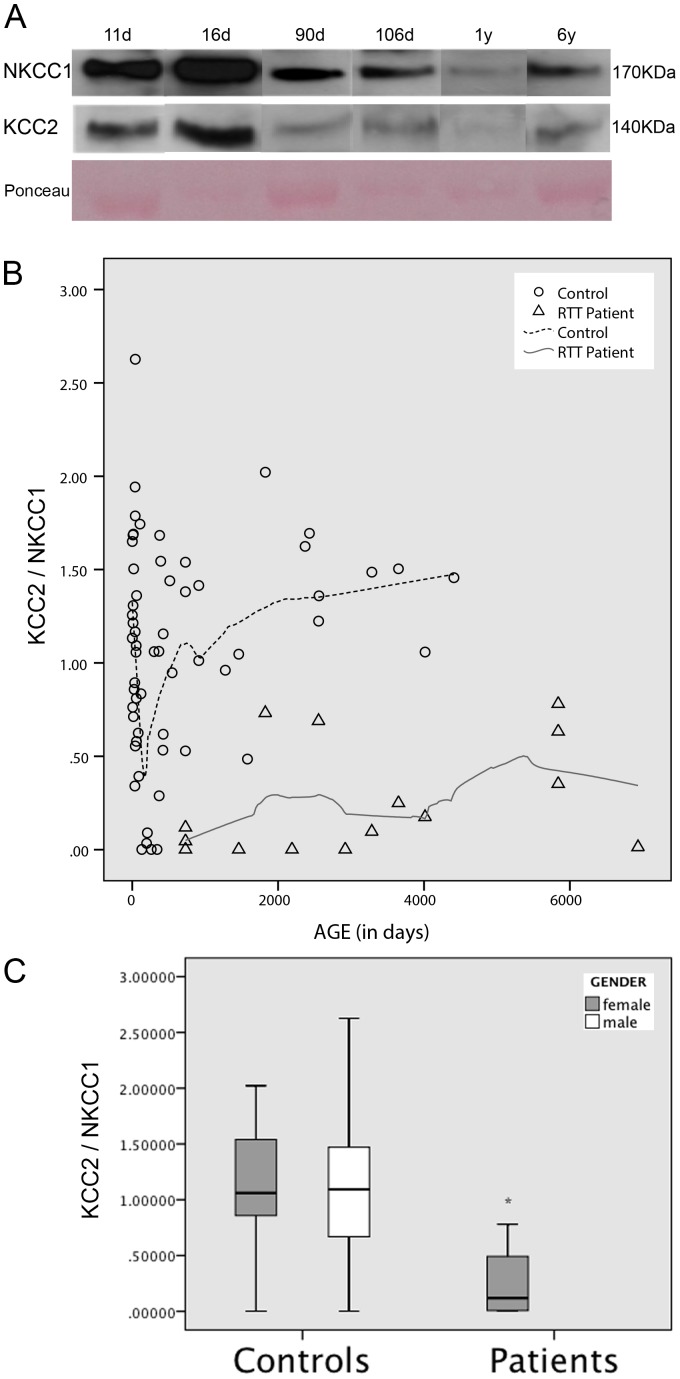
Cation Chloride Cotransporters ratio. (A) Immunoblot analysis of NKCC1 and KCC2. (B) Scatterplot of the relationship between Cotrasporters ratio (KCC2/NKCC1) and age for Rett Patients and Controls. Lines show a LOWESS smooth (locally-weighted polynomial regression - nonparametric smooth) suggesting a discrepant cotransporter ratio between Patients and Controls supported by the respective ANCOVA (*F* (1, 70) = 30.08, *p* = .001, *η_p_^2^* = .29). (C) Boxplot of Cotransporters ratio (KCC2/NKCC1) for Rett Patients and Controls (males and females). *p = 0.001.

To determine whether there were significant differences in the ratio of the two synaptic proteins (KCC2/NKCC1) on the CSF of the healthy population and RTT patients a between-subjects ANCOVA was performed, controlling for the effect of age and gender. This analysis revealed a statistically significant difference between Healthy controls and RTT patients, *F* (1, 70) = 26.56, *p<*.001, *η_p_^2^* = .28 even when controlling for the possible confounding effect of age and gender (figure 2B and 2C). RTT patients presented a significantly lower KCC2/NKCC1 ratio (*M* = .26, *SD* = .30) than Healthy controls (*M* = 1.08, *SD* = .56). Even if the effects of age and gender would not have been controlled for in the analysis the decrease in KCC2/NKCC1 ratio was still significant (*F* (1, 70) = 30.08, *p* = .001, *η_p_^2^* = .29).

## Discussion

In this study, we demonstrate reduced KCC2 levels and KCC2/NKCC1 ratio in the CSF of RTT patients. These findings suggest that altered inhibitory GABA function can underlie the pathophysiology of RTT and also play a role in the epileptogenesis of this neurodevelopmental disorder, in which epilepsy is present in around 70% of patients [3]. Detection of transmembrane synaptic proteins in the CSF is a useful tool in the study of neurotransmission disorders, as recently reported by our group [19]. Despite their low abundance compared to the global CSF proteome [25], the proteins here studied (NKCC1 and KCC2) were readily detected and at the expected molecular weight. Harrington and co-workers [26] identified the presence of CSF membranous nanostructures that can provide an appropriate environment for transmembrane proteins, which are hydrophobic in nature. Their morphology is similar to that of synaptic vesicles and exosomes; their structure resembles that of nanotubules, cell-to-cell interacting structures that facilitate the selective transfer of membrane vesicles and organelles but which seem to impede the flow of small molecules [27]. The intensity and resolution of the different bands obtained with the immunoblot procedure (Figures 1A and 2A) strongly supports the applicability of this analysis in neurochemical research. To our knowledge, this is the first report of detection of cation chloride cotransporters in CSF.

CSF turnover ratio and the extent of central nervous system cell death and synaptic pruning can influence circulating protein levels but these factors are likely to affect both proteins, NKCC1 and KCC2, equally. During the first months of postnatal life there is a period of intense synaptogenesis that subsequentially decreases. This is probably the cause for the reduction of protein levels in CSF observed during the first year of life. In fact, regarding transmembrane proteins like NKCC1 and KCC2, we have observed the same tendency that also was detected with other synaptic proteins [19]. The same phenomena could explain the fact that, in the CSF, both cation chloride cotransporters exhibit a reduction and KCC2 does not increase in the CSF, as was expected from previous studies in brain tissue [28], [29]. Experimental limitations in humans have been an obstacle in obtaining direct evidence of age-related changes in GABAA-R physiology [28].

In mice, loss of MeCP2 leads to reduced expression of BDNF after birth [15] and in humans evidence of BDNF reduction in RTT has also been detected [29]. The effects of BDNF on neurotransmission in developing and mature neurons have been partly associated with the regulation of GABAergic transmission. Apart from its effects on GABAergic innervation [30], BDNF can also promote the functional maturation of GABAA-R mediated responses by inducing upregulation of KCC2 [16], [17]. The imbalance between excitatory and inhibitory functions in RTT has been associated with reduced BDNF [31] and GABA levels, decreased expression of GABA receptor subunits [32], reduced expression of the enzymes glutamic acid decarboxylase 67 and glutamic acid decarboxylase 65 [8], reduced number of glutamatergic synapses [33] and reduced strength of basal inhibitory rhythms [34]. Moreover, exogenous BDNF has been shown to rescue synaptic dysfunction in Mecp-2 null mice. However, the mechanism by which reduced levels of BDNF contribute to disease and also to the phenotypical rescue is not completely understood [31].

Our results suggest an immature pattern of GABAergic neurotransmission in RTT patients, by revealing a dysregulation on the KCC2/NKCC1 ratio (the two major contributors to intracellular chloride concentration) and this evidence in humans is in accordance with the relevance of MeCP2 for GABAergic function described in animal models [8]. An imbalance between excitatory and inhibitory synaptic events, in the brain of children with neurodevelopmental disorders that have epilepsy and autism as key features, is a postulated general mechanism. Moreover, KCC2/NKCC1 ratio dysregulation is a particularly interesting specific molecular change, already described for diseases like tuberous sclerosis [35].

### Conclusions

We describe a significant decrease of KCC2 in the cerebrospinal fluid of Rett patients. A major advantage of doing these *in vivo* studies in children with severe neurologic disorders like RTT, is that it allows to search for disturbances in the normal developmental pattern. Therefore, our findings might have implications for the understanding of RTT pathophysiology, considering that KCC2 is a neuronal specific protein with a key role for neuronal electrical function and structure, properties that are known to be altered in *Mecp2* mutated neurons. Moreover, these results could bring light to new therapeutic approaches, particularly through the pharmacological manipulation of the cation chloride cotransporters. Further studies in the MECP2 knockout model and other models to study the disease process are needed to explore these possibilities.

## Supporting Information

Table S1 **Socio-demographic variable: mean values (and standard deviations) of age.** Significant differences among the groups were assessed with Mann-Whitney Test (*U*).
(DOCX)
