# Supplementary material for: Abnormal Expression of Cerebrospinal Fluid Cation Chloride Cotransporters in Patients with Rett Syndrome
Source: PLoS One. 2013 Jul 19;8(7):e68851. doi: 10.1371/journal.pone.0068851 (PMC3716803; doi:10.1371/journal.pone.0068851)
Supplement: Table S1 — Socio-demographic variable: mean values (and standard deviations) of age. Significant differences among the groups were assessed with Mann-Whitney Test (U). (DOCX) [file pone.0068851.s001.docx]

**Table S-1** – Socio-demographic variable: mean values (and standard deviations) of age. Significant differences among the groups were assessed with Mann-Whitney Test (*U*).

|  | **Rett Patients**  (n=16) | **Controls**  (n=65) | **Statistics** | ***p-value*** |
| --- | --- | --- | --- | --- |
| **Age** | 3444.69  (2173.59) | 746.00  (1075.89) | *U*=932.00 | *< .001* |
